# In Memoriam: Masahiko Sato (1932–2025)

**DOI:** 10.1186/s40101-026-00426-w

**Published:** 2026-05-30

**Authors:** Tetsuo Katsuura

**Affiliations:** Graduate School of Engineering, Chiba University, Chiba, Japan

Professor Masahiko Sato passed away on April 11, 2025, at Central Hospital in Tokyo at the age of 92. He was an outstanding physiological anthropologist and an exceptional educator who made seminal contributions to the establishment of physiological anthropology in Japan and to its subsequent development.

Professor Sato was born on December 4, 1932, in Kushiro, Hokkaido. He graduated from the Department of Biology, Faculty of Science, The University of Tokyo, in March 1957. In March 1962, he completed the doctoral program in anthropology at the Graduate School of the same university and simultaneously received the degree of Doctor of Science.

He began his academic career as an assistant professor at The University of Tokyo in April 1962. In April 1965, he became an associate professor at Japan Women’s College of Physical Education, and in April 1968, he was appointed professor at the newly established Kyushu Institute of Design. He retired in 1996 upon reaching the mandatory retirement age and was awarded the title of Professor Emeritus of Kyushu Institute of Design. After retirement, he continued his academic activities as a professor at Bunka Women’s University from April 1997 to March 2003 and as a professor at Nagasaki Junior College from April 2003 to March 2007.

My first encounter with Professor Sato occurred in February 1968, when I was a senior in high school. At that time, Kyushu Institute of Design held its inaugural recommendation-based entrance examination in Fukuoka. Upon entering the large classroom for the interview, I was greeted by a young professor holding a skeletal specimen of a human lower limb, who posed several questions to me. That professor was none other than Masahiko Sato. I still vividly recall that he was 35 years old at the time—youthful and dashing in appearance. In the latter half of my third year at the university, I chose Professor Sato’s laboratory without hesitation. Including the period during which I served as his assistant professor, I had the privilege of receiving his direct guidance for a total of 12 years. Since then, I have been a beneficiary of his immeasurable kindness and generosity, both professionally and personally.

Professor Sato’s path toward physiological anthropology appears to have begun with his encounters with Professor Toshihiko Tokizane of the Faculty of Medicine, The University of Tokyo. Professor Tokizane began offering a lecture titled “Physiological Anthropology” in 1954, which Professor Sato attended often, first as a student and later as an assistant professor. Professor Tokizane conducted a wide range of pioneering studies, including voice analysis, electromyography, the cerebral activation system, the limbic system, and cortical association areas. Through these studies, he produced numerous outstanding achievements and became a world authority in brain physiology and electromyography. Unfortunately, on August 3, 1973, Professor Tokizane suddenly passed away at the age of 63. Professor Sato received news of his mentor’s passing while conducting physiological anthropological research on *ama* (traditional female divers) on Iki Island, Nagasaki Prefecture. I remember how deeply he was affected by this loss.

Under Professor Tokizane’s mentorship, Dr. Sato constructed his own state-of-the-art electroencephalographs and electromyographs and conducted research on neurophysiology and electromyography. After moving to the Kyushu Institute of Design, he vigorously pursued studies on environmental adaptation using the Biotron (artificial climatic chamber), which was completed in 1971. This Biotron was one of the largest and most advanced research facilities of its kind worldwide. It enabled the precise control of environmental factors such as air temperature, humidity, airflow, illumination, atmospheric pressure, and oxygen concentration for both human and animal experiments. Research conducted using this facility has had a significant impact on physiological anthropology research in Europe and the United States and has contributed greatly to the subsequent development of physiological anthropology.

One notable achievement of Professor Sato’s work during this period was the establishment of a new method for estimating lower and upper critical temperatures, which are indicators of tolerance to cold and heat. The lower critical temperature is defined as the temperature below which homeothermic mammals increase their heat production to maintain thermal balance, whereas the upper critical temperature is the highest temperature at which the resting metabolic rate can be maintained without an increase due to elevated body temperature. These critical temperatures have long been considered sensitive indices for estimating the thermal adaptability of homeotherms.

Traditionally, the lower critical temperature was estimated using a simple approach known as the “intersect method,” and no established method existed for estimating the upper critical temperature. Professor Sato developed a new method using polynomial regression equations relating metabolic rate to ambient and body temperatures, together with conversion equations that allow metabolic rate values to be translated into temperature values. He termed this approach the “polynomial equation method.” These equations were derived from measurements of metabolic rate, skin temperature, and rectal temperature in subjects resting in a semi-supine position under ambient temperatures of 20 °C, 25 °C, 30 °C, 35 °C, 40 °C, 45 °C, and 50 °C, with relative humidity maintained at 50% under all conditions. This innovative method enabled highly precise estimations of cold and heat tolerance and significantly advanced research in physiological anthropology.

Professor Sato later extended his research to the study of light environments. He actively investigated not only the effects of brightness but also the influence of color temperature and wavelength, thereby opening new directions for the study of human physiological responses to light.

Professor Sato’s achievements are reflected not only in numerous academic papers published in Japan and abroad, but also in more than 40 books. Many of these works were written for a broader readership and introduced the concepts of physiological anthropology in an accessible and engaging manner. Among these are *Humans and Climate: An Approach from Physiological Anthropology* (1987, Chuokoron-shinsha), *The Constitution of the Japanese and Foreigners* (1988, Kodansha), *Why Are Japanese Noses Low? From the Perspective of Physiological Anthropology* (1990, Nikkei Inc.) and *Encyclopedia of the Body* (2009, Maruzen). These publications have contributed greatly to the dissemination of physiological anthropology in Japan, although their influence has been limited internationally due to their publication in Japanese.

Professor Sato also played a central role in establishing the institutional foundations for physiological anthropology in Japan. In November 1978, along with 50 members of the Anthropological Society of Nippon, he founded the Study Group of Physiological Anthropology, which later developed into the Japanese Society of Physiological Anthropology. The society has grown steadily, and its English-language journal, *Journal of Physiological Anthropology*, has become an important platform for international academic exchange in the field and now possesses a high impact factor. Professor Sato served as a leading figure in the society and as its president from June 1993 to May 2007.

In addition to his contributions to Japan, Professor Sato has actively promoted international collaboration in physiological anthropology. Through his extensive international network, he has developed close collaborations with leading researchers, including Hans W. Jürgens (Kiel University), Pavao Rudan (Croatian Academy of Sciences and Arts), Elena Godina (Moscow State University), C. G. N. Mascie-Taylor (University of Cambridge), Alan H. Bittles (Edith Cowan University), and Douglas E. Crews (The Ohio State University). These international exchanges contributed to the organization of the first International Congress of Physiological Anthropology (ICPA), held in Tokyo in 1991. Since then, the congress has been held approximately every 2 years in different countries and has become an important forum for international research exchanges in the field. To further promote global collaboration, Professor Sato also played a pioneering role in establishing the International Association of Physiological Anthropology (IAPA) in 1996, thereby advancing the internationalization of the discipline.

Professor Sato was widely respected as an educator. During his 28 years at the Kyushu Institute of Design, he supervised more than 100 students and trained many researchers who later became university professors and industry leaders. Even in introductory lectures for first-year students, he offered intellectually demanding classes using numerous original English-language research papers. He consistently encouraged his students to aim for the highest standards of scholarship, often reminding them that their true competitors were scholars at the world’s leading universities and urging them to pursue excellence. At the same time, he maintained warm personal relationships with his students, frequently inviting members of his laboratory to his home and organizing mountain trips throughout the Kyushu region.

In recognition of his distinguished achievements, Professor Sato was awarded the Order of the Sacred Treasure, Gold Rays with Neck Ribbon, in 2011. In March 2013, he received the International Tokizane Prize in Physiological Anthropology.

The field of physiological anthropology that Professor Sato helped establish continues to develop steadily both in Japan and internationally. His philosophy of research and education will undoubtedly be carried forward by future generations.

We sincerely pray for the peaceful repose of Professor Sato’s soul.


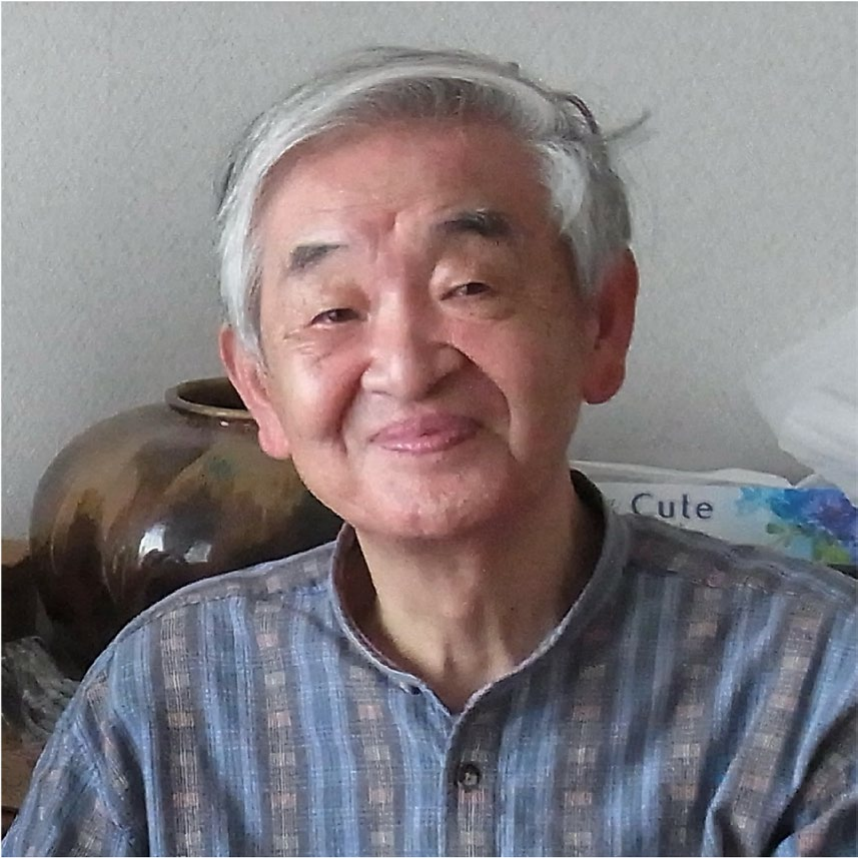


**Figure:**
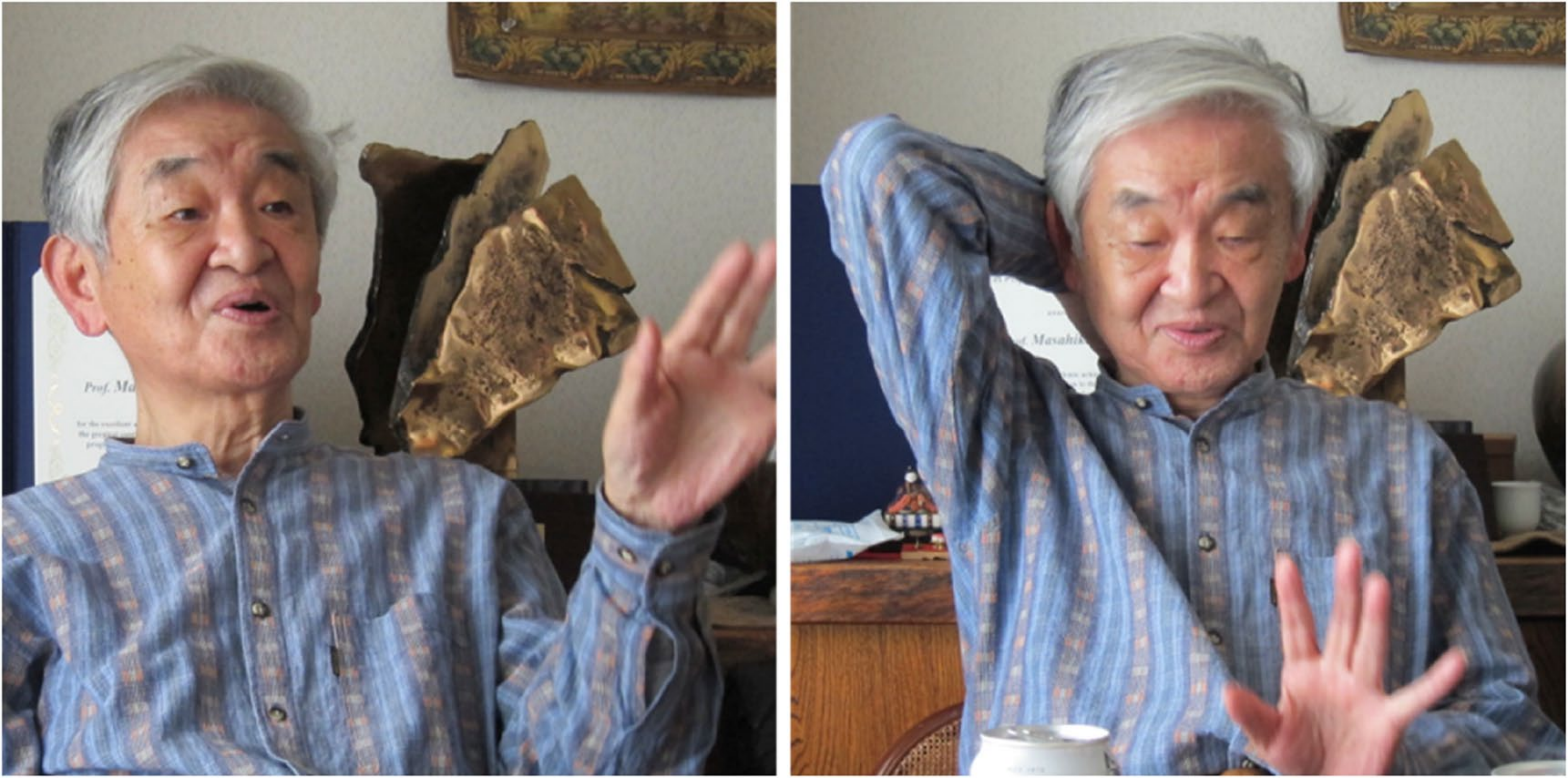
Professor Masahiko Sato passionately discussing the field of physiological anthropology.

